# Comprehensive analysis of the KSHV MiRNA targetome by Ago-HITS-CLIP

**DOI:** 10.1186/1750-9378-7-S1-O4

**Published:** 2012-04-19

**Authors:** Irina Haecker, Lauren Gay, Alison Morse, Marty McCrory, Yajie Yang, Jianhong Hu, Lauren McIntyre, Rolf Renne

**Affiliations:** 1 Department of Molecular Genetics and Microbiology, University of Florida, Gainesville, FL, USA

## 

The gamma-herpesvirus Kaposi’s Sarcoma-associated Herpesvirus (KSHV) is the etiological agent of Kaposi’s Sarcoma (KS), Primary Effusion Lymphoma (PEL) and a subset of Multicentric Castleman’s Disease (MCD) in immunocompromised individuals. As all herpesviruses KSHV has a latent and a lytic life cycle. Malignant cells in KS, PEL, and MCD are latently infected with KSHV. Interestingly, the virus encodes 12 miRNA genes giving rise to 25 mature miRNAs that are predominantly expressed during latency, i.e. in malignant cells. This, together with the increasing evidence for the involvement of miRNAs in cancer, suggests a potential role for the KSHV miRNAs in viral tumorigenesis. However, to date, very little is known about the function of these viral miRNAs. To address the question we performed Ago-HITS-CLIP [1] using the anti-Ago antibody 2A8 [2] to isolate RISC complexes from KSHV-infected lymphoma cells (BCBL1, BC3). RNAs extracted from these complexes were analyzed by Illumina sequencing to identify viral and cellular miRNAs and their target genes. The search for canonical seed sequence matches (nt 2-7) of the KSHV miRNAs within the mRNA-derived sequencing tags revealed more than 1000 cellular targets. Gene ontology analysis revealed that KSHV miRNA targets are enriched in genes involved in apoptosis, lymphocyte activation, cell cycle regulation, and transcriptional control. Importantly, we reproducibly obtained clusters of reads on experimentally confirmed KSHV miRNA target sites in several known target genes (e.g. Bach1, BCLAF1, and THBS1). New target genes are in the process of experimental validation, with 4 confirmed targets so far: TP53INP1, TPD52, ANXA2, C/EBPB. Target analysis for non-canonical seeds as well as for cellular miRNAs is currently ongoing.
